# Therapeutical approaches to paroxysmal hemicrania, hemicrania continua and short lasting unilateral neuralgiform headache attacks: a critical appraisal

**DOI:** 10.1186/s10194-017-0777-3

**Published:** 2017-07-20

**Authors:** Carlo Baraldi, Lanfranco Pellesi, Simona Guerzoni, Maria Michela Cainazzo, Luigi Alberto Pini

**Affiliations:** Medical Toxicology - Headache and Drug Abuse Centre, University of Modena and Reggio Emilia, Via del Pozzo 71, 41124 Modena, Italy

## Abstract

**Background:**

Hemicrania continua (HC), paroxysmal hemicrania (PH) and short lasting neuralgiform headache attacks (SUNCT and SUNA) are rare syndromes with a difficult therapeutic approach. The aim of this review is to summarize all articles dealing with treatments for HC, PH, SUNCT and SUNA, comparing them in terms of effectiveness and safety.

**Methods:**

A survey was performed using the pubmed database for documents published from the 1st January 1989 onwards. All types of articles were considered, those ones dealing with symptomatic cases and non-English written ones were excluded.

**Results:**

Indomethacin is the best treatment both for HC and PH. For the acute treatment of HC, piroxicam and celecoxib have shown good results, whilst for the prolonged treatment celecoxib, topiramate and gabapentin are good options besides indomethacin. For PH the best drug besides indomethacin is piroxicam, both for acute and prolonged treatment. For SUNCT and SUNA the most effective treatments are intravenous or subcutaneous lidocaine for the acute treatment of active phases and lamotrigine for the their prevention. Other effective therapeutic options are intravenous steroids for acute treatment and topiramate for prolonged treatment. Non-pharmacological techniques have shown good results in SUNCT and SUNA but, since they have been tried on a small number of patients, the reliability of their efficacy is poor and their safety profile mostly unknown.

**Conclusions:**

Besides a great number of treatments tried, HC, PH, SUNCT and SUNA management remains difficult, according with their unknown pathogenesis and their rarity, which strongly limits the studies upon these conditions. Further studies are needed to better define the treatment of choice for these conditions.

**Electronic supplementary material:**

The online version of this article (doi:10.1186/s10194-017-0777-3) contains supplementary material, which is available to authorized users.

## Background

Trigeminal autonomic cephalalgias (TACs) is a rare group of headaches characterized by unilateral attacks of severe throbbing pain, mainly localized in the orbital region, associated with unilateral cranial autonomic signs such as lacrimation, conjunctival injection, palpebral ptosis, rhinorrhoea, eyelid edema, facial sweating, facial redness and ear-fullness. The International Classification of Headache Disorders 3rd Edition beta version (ICHD-III-beta) recognizes 4 TACs: cluster headache (CH), hemicrania continua (HC), paroxysmal hemicrania (PH) and short-lasting unilateral neuralgiform headache attacks (SUNCT and SUNA) [1]. HC is characterized by a continuous background of moderate pain intensity and has only recently been classified as a TAC [2]; on the contrary, CH, PH, SUNCT and SUNA lack the history of background pain [1]. TACs rather than CH are uncommon and neglected syndromes: the annual prevalence of PH and short lasting unilateral neuralgiform headache attacks is about 0.5/1000 in the general population and is still unknown for HC [3], this facilitate their misdiagnosis, which often delays the correct treatment [4]. Treatment delay, especially in chronic forms, dramatically decreases the patients’ quality of life because pain is often severe, highly-disabling and can last, even if not continuously, for many hours during the day [5]. Only a few therapeutic tools are available for these conditions and this is firstly due to their infrequent diagnosis, which makes the conduction of well-prepared randomized clinical placebo-controlled trials (RCPCTs) almost impossible. The effectiveness and safety of the treatments are reported mainly in case-reports, case-series, letters to the editor and brief communications. This leads to a not-scheduled treatment for TACs and the absence of shared guidelines. Furthermore, there aren’t studies clearly ranking treatments to manage TACs, nor one comparing them in terms of effectiveness and/or safety. The aim of this study is to rank all therapeutic options available in literature for HC, PH, SUNCT and SUNA treatment and to compare, when possible, their effectiveness and safety. Since there are already shared guide-lines and a large amount of reviews dealing with CH, this won’t be discussed further.

## Methods

### Search strategy

A MEDLINE search using the electronic data-base pubmed has been performed to check all articles dealing with the treatment of primary HC, PH, SUNCT and SUNA form the 1st of January 1989 (the first complete year in which the first International Headache Society classification was available) onwards. All articles types were considered and non-English written ones were excluded. The research was performed using the following terms: “((paroxysmal hemicrania) AND (“1989/01/01”[Date - Publication]: “3000”[Date - Publication])) AND English[Language]” for PH, “((hemicrania continua) AND (“1989/01/01”[Date - Publication]: “3000”[Date - Publication])) AND English[Language]” for HC, “((short lasting neuralgiform headache attacks) AND (“1989/01/01”[Date - Publication]: “3000”[Date - Publication])) AND English[Language]” for SUNCT and SUNA. Short lasting unilateral neuralgiform headache attack was treated as one entity, not differentiating between short lasting neuralgiform headache attacks with conjunctival injection and tearing (SUNCT) and short lasting neuralgiform headache attacks with autonomic signs (SUNA). A few articles cited in the references of the above-mentioned ones were cited even though they were not present in pubmed, but were found in SCOPUS and EMBASE.

### Data

**Fig. 1:**
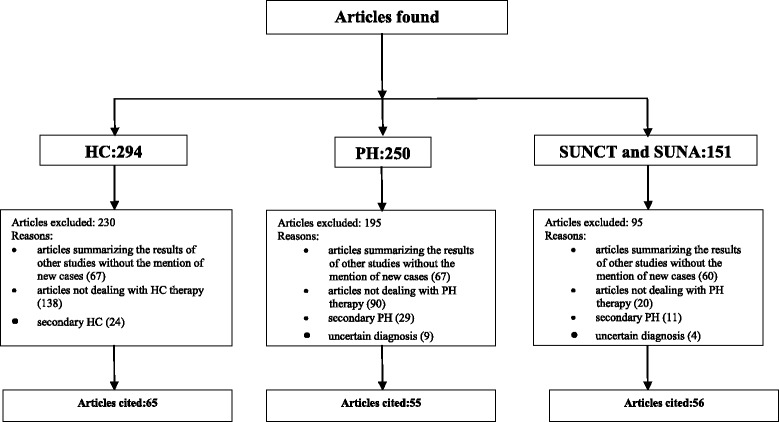
Flow-chart of article selection

Altogether, 691 articles were found of which 290 articles for HC, 250 for PH and 151 for short lasting unilateral neuralgiform headache attacks. Cited articles should fulfill the ICHD-III beta guide-lines for TACs diagnosis, not deal with a symptomatic case and correctly state treatment. Reviews were considered only if new cases were included. For HC, 230 articles were excluded: 67 summarized results from other studies without adding any new case, 138 didn’t deal with HC therapy and 24 referred to symptomatic cases. For PH, 195 articles were excluded: 67 reported and summarized only the results of different works, 90 didn’t consider PH therapy or described it unsatisfactorily, 29 referred to symptomatic PH and 9 didn’t fulfill all ICHD-III diagnostic criteria, making a diagnosis of “probable PH”. For SUNCT and SUNA 95 articles were excluded: 60 were reviews, 20 of them didn’t deal with SUNCT or SUNA therapy or reported it unsatisfactorily, 11 reported symptomatic cases and 4 didn’t full-filled all diagnostic criteria. Steps followed for article selection are summarized in Fig. 1. For every article, each patient was analyzed and only those treatments correctly stated in terms of regimen and response were considered. If a patient took a drug in different dosages or underwent a non-pharmacological procedure following different regimens, only the one giving the maximum effect was considered. Every patient was classified as a responder if he/she was accredited with, at least, a partial relief. Moreover, as to grade the different therapies better, pain-free patients were sub-classified as complete responders. Finally, the signaled AEs were collected. Since all these diseases are characterized by exacerbations periods in which pain attacks develops and inter-critic periods in which pain is absent (PH, SUNCT and SUNA) or slight-moderate (HC), treatments were divided in two categories: treatments used to cease attacks during exacerbations and treatments taken regularly to control pain (especially in HC), trying to prevent the incoming of new active phases. The first treatments were indicated as “acute treatments”, whilst the second as “prolonged treatments”. Some acute treatments in HC and PH were used also to control pain outside exacerbations and were both considered as acute and prolonged treatments.

Drug mean dosage and therapeutic standards for non-pharmacological treatments were considered and summarized, even if not statistically analyzed.

Treatments used in less than five patients or which were clearly ineffective were not pooled in the statistical analysis, even if reported. Data regarding treatments used in 5 or more patients are summarized in Table 1, those ones regarding treatments used in less than 5 patients are reported in the Additional file 1: Table S1.

**Table 1:** Treatment options for HC, PH and SLUNHA used in, at least, 5 patients

Treatment	Number of patients	Mean dosage ±SD* [range]	Route of administration	Responders proportion % [95% CI]	Complete responders proportion % [95% CI]	AE proportion % [95% CI]	AE causing the stoppage or reduction of therapy proportion % [95% CI]	References
Section A- Hemicrania continua								
*Acute treatments*								
Indomethacin	159	Adult: 145 ± 125 [25–325] Pediatric: 100 ± 50 [25–175]	IM 1.3% REC 0.6% OS 98.1%	157/159 99 [97–100]	151/159 95 [92–98]	75/83 90	46/83 55	[6–61]
SONB	17	**		17/17 100	5/17 29 [8–50]	-	-	[62–64]
GONB	15	***		6/15 40 [15–65]	1/15 7 [0–19]	-	-	[10, 35, 43, 48, 62]
Celecoxib	11	528 ± 241 [200–800]	OS 100%	9/11 82 [59–100]	8/11 73 [46–100]	-	-	[32, 49, 52, 65]
Piroxicam	7	37 ± 10 [20–40]	OS 100%	6/7 86 [60–100]	5/7 71 [38–100]	-	-	[40, 66]
MONB	6	0.5–1.5 mg/ml solution with 12.μrg/m andrenaline		0/6 0	0/6 0	-	-	[62]
Oxygen	13	8 ± 5^a^	INAL 100%	0/13 0	0/13 0	-	-	[39, 47]
Sumatriptan	8	6	SC 100%	0/7 0	0/7 0	-	-	[32, 67]
*Prolonged treatments*								
Indomethacin	159	Adult:115 ± 100 [25–225] Pediatric:55 ± 35 [25–75]^c^	IM 1.3% REC 0.6% OS 98.1%	157/159 99 [97–100]	151/159 95 [92–98]	75/83 90	46/83 55	[6–61]
SONB	17	**		17/17 100	5/17 29 [8–50]	-	-	[62–64]
Melatonin	17	12 [3–30]	OS 100%	9/17 53 [29–77]	5/17 29 [8–50]	6/13 45	3/13 23	[13, 21, 31, 33, 37, 48]
GONB	15	***		6/15 40 [15–65]	1/15 7 [0–19]	-	-	[10, 35, 43, 48, 62]
ONS	14	****		12/14 84 [68–100]	3/14 21 [3–39]	-	-	[10, 33, 68]
Gabapentin	13	1600 [600–3600]	OS 100%	11/13 85 [65–100]	6/13 46 [19–73]	4/9 44	0/9 0	[7, 21, 32, 43, 55, 69]
Topiramate	13	133 [50–300]	OS 100%	11/13 85 [65–100]	8/13 62 [35–89]	2/7 29	2/7 29	[11, 24, 28, 29, 36, 38, 43, 49, 70]
OnabotulinumtoxinA	12	155^b^ [100–185]	SC 100%	12/12 100	4/12 33 [6–60]	-	-	[22, 43]
Celecoxib	11	528 ± 241 [200–800]	OS 100%	9/11 82 [59–100]	8/11 73 [46–100]	-	-	[32, 49, 52, 65]
Verapamil	8	265 [120–480]	OS 100%	3/8 38 [4–72]	0/8 0	1/1 100	1/1 100	[7, 21, 32, 43, 55, 69]
Piroxicam	7	37 ± 10 [20–40]	OS 100%	6/7 86 [60–100]	5/7 71 [38–100]	-	-	[40, 66]
MONB	6	0.5–1.5 mg/ml solution with 12.μrg/m andrenaline		0/6 0	0/6 0	-	-	[62]
Section B- Paroxysmal hemicrania								
*Acute treatments*								
Indomethacin	168	Adult: 97 ± 39 Pediatric: 35 ± 27	OS 95% IM 0.6% RECTAL 4.4%	163/168 97 [94–100]	150/168 89 [85–94]	42/78 54 [43–64]	21/78 27 [17–37]	[26, 38, 53, 60, 71–118]
Sumatriptan	24	6	SC 100%	5/24 21 [5–37]	1/24 4 [0–8]	1/1 100	1/1 100	[38, 76, 84, 103, 104, 114]
Oxygen	11	7 ± 4^a^	INAL 100%	6/18 33 [11–55]	0/18 0	-.	-	[38, 89, 119]
SONB	6	**		0/6 0	0/6 0	-	-	[62]
GONB	6	**		0/6 0	0/6 0	-	-	[62]
MONB	6	**		0/6 0	0/6 0	-	-	[62]
Piroxicam	5	36 ± 9	OS 100%	3/5 60 [17–100]	2/5 40 [0–80]	-	-	[66]
*Prolonged treatments*								
Indomethacin	168	Adult: 97 ± 39 Pediatric: 35 ± 27^c^	OS 95% IM 0.6% RECTAL 4.4%	163/168 97 [94–100]	150/168 89 [85–94]	42/78 54 [43–64]	21/78 27 [17–37]	[26, 38, 53, 60, 70–118]
Verapamil	30	Adult: 248 ± 87 Pediatric: 200 ± 70	OS 100%	14/30 47 [26–64]	5/30 17 [3–31]	2/3 66	1/3 33	[38, 81, 83, 87, 91, 92, 98, 101, 103, 111, 115]
Carbamazepine	15	803 ± 275	OS 100%	3/15 20 [0–40]	0/15 0	-	-	[84, 98, 101, 107, 110, 120]
Topiramate	12	Adult: 172 ± 75 Pediatric: 48 ± 3	OS 100%	9/12 75 [50–99]	5/12 42 [14–70]	2/2 100	2/2 100	[38, 75, 93, 101, 105, 109, 115]
SONB	6	**		0/6 0	0/6 0	-	-	[62]
GONB	6	**		0/6 0	0/6 0	-	-	[62]
MONB	6	**		0/6 0	0/6 0	-	-	[62]
Piroxicam	5	36 ± 9	OS 100%	3/5 60 [17–100]	2/5 40 [0–80]	-	-	[66]
Amitriptyline	5	32 ± 17	OS 100%	2/5 40 [0–80]	0/5 0	-	-	[73, 78, 91, 92]
Section C- Short lasting unilateral neuralgiform headache attacks								
* Acute treatments*								
Lidocaine	36	1.9 [1–3.5]	IV 75% SC 25%	34/36 94 [87–100]	29/36 80 [67–93]	13/36 36 [15–58]	6/36 16 [1–31]	[124–129]
Prednisone	11	53 [20–100]	OS 91% IV 9%	6/11 50 [20–80]	1/11 10 [0–28]	-	-	[124, 129–134]
Methylprednisolone	7	193 [16–1000]	IV 57% OS 43%	5/7 71 [38–100]	4/7 57 [20–94]	-	-	[125, 132, 135–137]
Phenytoin	5	270 [200–300]	OS 100%	1/5 20 [0–55]	0/5 [0–0]	-	-	[124, 138–141]
*Prolonged treatments*								
Lamotrigine	84	231 [50–900]	OS 100%	68/84 81 [73–89]	38/84 45 [35–55]	32 [16–48]	13 [12–25]	[124, 125, 127–129, 134, 139, 140, 142–155]
Carbamazepine	78	737 [100–2000]	OS 100%	38/78 49 [38–60]	9/78 11 [4–18]	50 [20–80]	40 [10–70]	[124–127, 131–134, 137–140, 143, 146, 147, 150–152, 155–157, 159–166]
Indomethacin	50	116 [50–225]	OS 100%	4/50 8 [1–15]	1/50 2 [0–4]	50 [0–100]	-	[124, 125, 127, 129, 131, 134, 138, 139, 141, 146–148, 150, 154, 157–159, 162, 165, 167–171]
Gabapentin	48	1581 [300–3600]	OS 100%	28/48 59 [45–74]	13/48 28 [15–41]	0/8 0	0/8 0	[124–127, 137, 138, 140, 141, 145, 150, 153, 167, 172, 173]
Topiramate	36	168 [40–400]	OS 100%	20/36 56 [39–72]	10/36 28 [13–43]	75 [32–100]	2 [0–55]	[124–126, 129, 134, 135, 138, 143, 145, 148, 155]
VTA DBS	9	Amplitude: 4 mV Frequency: 185 Hz Pulse width: 60 ms		9/9 100	9/9 100	9/9 100	1/9 11	[174]
GONB	9	Bupivacaine 12.5 every 3 months		5/9 55 [44–66]	2/9 22 [33–44]	-	-	[125, 140]
ONS	7	Amplitude: 0.3–3.15 V Frequency: 60–130 Hz Pulse width: 450 ms		7/7 100	7/7 100	0/7 0	0/7 0	[175]
Verapamil	6	347 [240–640]	OS 100%	2/6 33 [0–71]	1/6 17 [0–34]	-	-	[134, 138, 139, 162, 170]
Valproate	5	950 ± 655 [250–2000]	OS 100%	0/5 0	0/5 0	-	-	[124, 131, 139, 168]

*For non-pharmacological procedures the method used has been reported. Drug dosages are in mg/day if not otherwise specified

**Antonaci: 0.5–1.5 mg/ml solution with 12.5 μg/m andrenaline; Guerrero 2 cm^3^ of 0.5% bupivacaine and 2% mepivacaine in a 1:1 ratio¸ Weyker 25% 0.25 ml + bupivacaine 10 mg triamcinolone

***Beams: 9 cm^3^ of 1% lidocaine with 40 mg triamcinolone; Garza and Guerrero: 2 cm^3^ of 0.5% Bupivacaine and 2% mepivacaine in a 1:1 ratio

****Burns: frequency of 60 Hz and pulse width of 250 μs for all patients; the amplitude of the bion current could be adjusted within a given range

^a^L/min, ^b^UI; ^c^maintainance dose, unchanged for, at least, 1 month

### Statistical analysis

Continuous data were expressed as mean ± standard deviation. Binary variables were express as proportion and percentages. Odds and odds ratios (OR) were considered for statistical analysis. Continuous data and odds were approximated at the second decimal figure, OR and all *p*-values at the third. Statistical analysis was performed using the STATAIc 13 software. For every syndrome, the odds of responders, complete responders, AEs and AEs causing treatment reduction or discontinuation were compared based on the test of the equality of odds.

## Results

### Hemicrania continua (HC)

Globally, 65 articles were considered for the statistical analysis [6–70]. Indomethacin was referred to as the most widely used treatment for HC. Melatonin was used in 17 patients, gabapentin and topiramate were utilized in 13 patients, onabotulinumtoxinA (OnabotA) in 12 patients and celecoxib in 11 patients. The other drugs were used in less than 10 patients. Supraorbital nerve blockade (SONB) was used on 17 patients, great occipital nerve blockade (GONB) on 15, occipital nerve stimulation (ONS) on 14 patients and minor occipital nerve blockade (MONB) on 6 patients.

Other drugs rather than indomethacin were used before indomethacin was given in 60% of cases, but only in the 20% of cases data were good enough to be considered (data not shown). Alternatively, since indomethacin was stopped in the 30% of cases because of its related AEs, other treatments were tried. Pharmacological treatments used in at least 5 patients, are summarized in Table 1 (section A). Statistical comparisons between the odds of responders and complete responders are summarized in Table 2 for the acute treatments and in Table 3 for the prolonged treatments. Data regarding those treatments performed in less than 5 patients are reported in Additional file 1: Table S1 (section A).

**Table 2:**
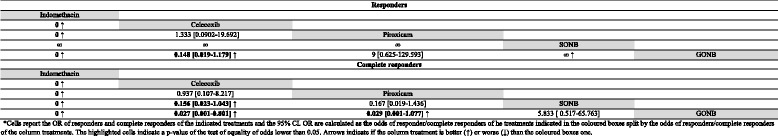
comparisons between the odds of partial and complete responders for the acute treatments of HC*

*Cells report the OR of responders and complete responders of the indicated treatments and the 95% CI. OR are calculated as the odds of responder/complete responders of he treatments indicated in the coloured boxes split by the odds of responders/complete responders of the column treatments. The highlighted cells indicate a *p*-value of the test of equality of odds lower than 0.05. Arrows indicate if the column treatment is better (↑) or worse (↓) than the coloured boxes' ones

**Table 3:**
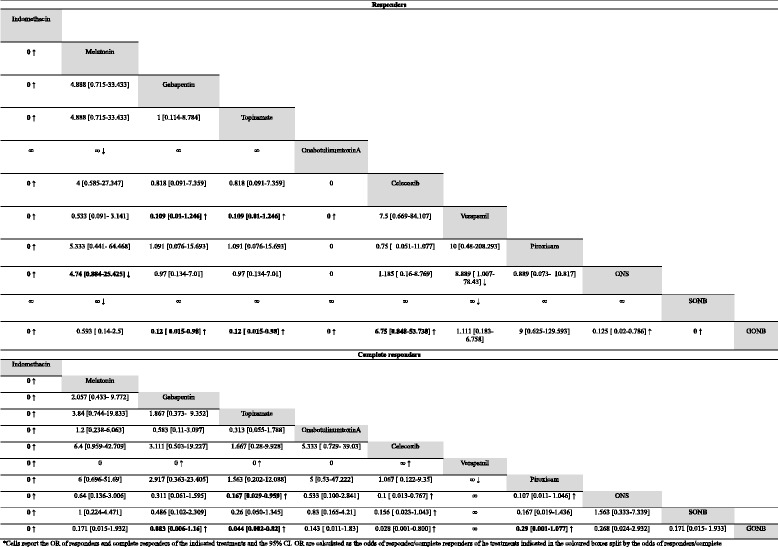
comparisons between the odds of responders and complete responders of prolonged treatments for HC*

*Cells report the OR of responders and complete responders of the indicated treatments and the 95% CI. OR are calculated as the odds of responder/complete responders of he treatments indicated in the coloured boxes split by the odds of responders/complete responders of the column treatments. The highlighted cells indicate a *p*-value of the test of equality of odds lower than 0.05. Arrows indicate if the column treatment is better (↑) or worse (↓) than the coloured boxes' ones

#### Effectiveness

##### Acute treatments

Indomethacin, supraorbital nerve blockade (SONB), great occipital nerve blockade (GONB), celecoxib, piroxicam, minor occipital nerve blockade (MONB), oxygen, sumatriptan, methylprednisolone, ibuprofen, dorsal root ganglion blockade (DRGB), sphenopalatine ganglion blockade (SPGB) and ergotamine were the drugs considered for exacerbation management in HC.

Oxygen, minor occipital nerve blockade (MONB) and sumatriptan seemed to have no effect on HC and no responders have been registered; for this reason they weren’t pooled in the statistical analysis. Ergotamine, ibuprofen, DRGB, SPGB and methylprednisolone weren’t pooled in the statistical analysis because of the small number of patients treated with these. Indomethacin has a significantly higher odds of responders than celecoxib (*p* < 0.001), piroxicam (*p* < 0.001) and GONB (*p* < 0.001), but a similar proportion of responders than SONB, which reduced painful symptoms in each patient (*p* = 0.541). Indomethacin has also the highest odds of complete responders, even if compared with SONB (all *p* < 0.001). Considering other treatments rather than indomethacin, piroxicam and celecoxib haven’t shown a significantly different odds of responders (*p* = 0.837) and complete responders (*p* = 0.219). Celecoxib has a higher odds of responders than GONB (*p* = 0.037) and a significantly higher odds of complete responders than GONB (*p* < 0.001) and SONB (*p* = 0.028). Finally, SONB shows a significantly higher odds of responders than GONB (*p* < 0.001), but a similar odds of pain-free patients (*p* = 0.105). All comparisons are summarized in Table 2.

##### Prolonged treatments

Indomethacin, melatonin, gabapentin, topiramate, OnabotA, celecoxib, verapamil, piroxicam, ONS, SONB, GONB, acemethacin, amytriptiline, DRGB, SPGB, valproate, lithium, troclear injections of triamcinolone, fentanyl and tilidine are the drugs used for the treatment of HC outside exacerbations, to prevent the incoming of new active phases and control the background pain. Data regarding acemethacin, amytriptiline, DRGB, SPGB, valproate, lithium, troclear injections of triamcinolone, fentanyl and tilidine were not pooled in the statistical analysis because of the small number of patients who tried them.

Indomethacin has a significantly higher odds of responders than all other treatments except for OnabotA (*p* = 0.723) and SONB (*p* = 0.541); moreover, it has a significantly higher odds of pain-free patients compared to the other types of treatment (all *p* < 0.001).

Considering the other types of treatment, verapamil has a lower odds of responders than gabapentin (*P* = 0.03), topiramate (*p* = 0.03), OnabotA (*p* = 0.002), ONS (*p* = 0.018) and SONB (*p* < 0.001). Verapamil has also a lower odds of complete responders than gabapentin (*p* = 0.027), topiramate (*p* = 0.006), celecoxib (*p* = 0.002) and piroxicam (*p* = 0.005). GONB has an odds of responder lower than gabapentin (*p* = 0.018), topiramate (*p* = 0.018), OnabotA (*p* = 0.001), celecoxib (*p* = 0.037), ONS (*p* = 0.008) and SONB (*p* < 0.001). Furthermore, it has a lower odds of complete responders than gabapentin (*p* = 0.018), topiramate (*p* = 0.002), celecoxib (*p* < 0.001) and piroxicam (*p* = 0.002).

Furthermore, melatonin has an odds of responders significantly lower than OnabotA (*p* = 0.006). All comparisons are summarized in Table 3.

#### Safety

Considering the poor number of signaled AEs, no statistical comparisons were made between the different odds of AEs and AEs causing the discontinuation or the modification of therapy. The only mild-quality data dealing with drugs’ safety profile regarded indomethacin: AEs status was clearly declared in 83 patients, 75% of whom reported an AE and 46 were forced to discontinue or reduce therapy.

### Paroxysmal hemicrania (PH)

Fifty five articles were considered for PH [26, 38, 53, 60, 62, 66, 71–123]. Indomethacin is the most used treatment (168 patients), followed by verapamil (30 patients), sumatriptan (24 patients) and oxygen (18 patients). Carbamazepine (CBZ) was tried on 15 patients, topiramate on 12 patients, amitriptyline and piroxicam on 5 patients. SONB, MONB and GONB were all used upon 6 patients. Piroxicam and amitriptyline were used upon 5 patients. All other treatments were used on less than 5 patients and were not taken into consideration for the statistical analysis. Treatments used in 5 or more patients are summarized in Table 1 (section B). Statistical comparisons of the odds of responders and complete responders for acute treatments are summarized in Table 4 whilst for the prolonged ones in Table 5. Data regarding those drugs taken by less than 5 patients are summarized in the Additional file 1: Table S1 (section B).

**Table 4:**
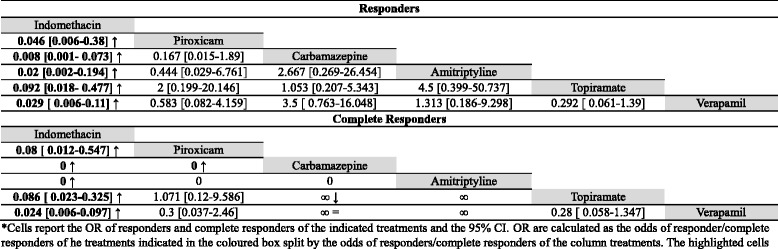
statistical comparisons between the odds of responders and complete responders of acute treatments for PH*

*Cells report the OR of responders and complete responders of the indicated treatments and the 95% CI. OR are calculated as the odds of responder/complete responders of he treatments indicated in the coloured box split by the odds of responders/complete responders of the column treatments. The highlighted cells indicate a *p*-value of the test of equality of odds lower than 0.05. Arrows indicate if the column treatment is better (↑) or worse (↓) than the coloured boxes' ones

**Table 5:**
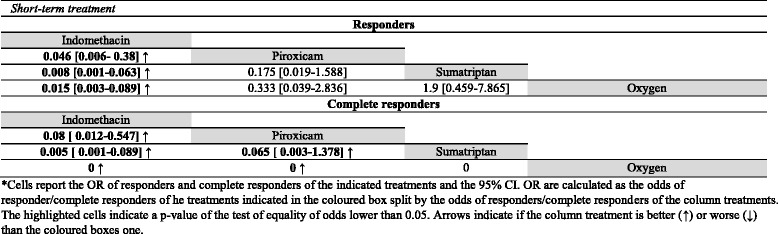
statistical comparisons between the odds of responders and complete responders of prolonged treatments for PH*

*Cells report the OR of responders and complete responders of the indicated treatments and the 95% CI. OR are calculated as the odds of responder/complete responders of he treatments indicated in the coloured box split by the odds of responders/complete responders of the column treatments. The highlighted cells indicate a *p*-value of the test of equality of odds lower than 0.05. Arrows indicate if the column treatment is better (↑) or worse (↓) than the coloured boxes' ones

#### Effectiveness

##### Acute treatments

Indomethacin, sumatriptan, oxygen, MONB, GONB, SONB, piroxicam, rofecoxib, prednisone, valdecoxib, etoricoxib, naproxen, betamethasone, methylprednisolone, HDBS and SPGB were considered as acute treatments. The last eight were used in less than 5 patients and so weren’t pooled in the statistical analysis; MONB, GONB and SONB weren’t pooled in the statistical analysis either as they were clearly ineffective. Rofecoxib was not considered as it has been taken off the International market. Indomethacin has a significantly higher odds of responders and complete responders than piroxicam, sumatriptan and oxygen (all *p* < 0.001). Moreover, piroxicam has a significantly higher odds of complete responders, both than sumatriptan (*p* = 0.0187) and oxygen (*p* = 0.006). All comparisons are reported in Table 4.

##### Prolonged treatments

To prevent the recurrence of PH exacerbations 26 treatments were find out from literature. Indomethacin, verapamil, CBZ, topiramate, MONB, GONB, SONB, piroxicam and amytriptiline were those treatments used in more than 5 patients and pooled in the statistical analysis. Propranolol, acetylsalicylic acid, lithium, ergotamine, dipyrone, valproate, acetazolamide, baclofen, phenytoin, methysergide, doxepine, flunnarizine, gabapentin, bethametasone, methylprednisolone, OnabotA, hypothalamic deep brain stimulation (HDBS), sphenopalatine ganglion blockade (SPGB) were used in less than 5 patients and so weren’t taken into consideration for the statistical analysis. Indomethacin has a the highest odds of responders and complete responders (all *p* < 0.001). Besides indomethacin, all other drugs show a not-significantly different odds of responders between them. Considering the complete responders, CBZ has a lower odds than piroxicam (*p* = 0.012) and topiramate (*p* = 0.007). All comparisons are reported in Table 5.

#### Safety

AEs were cited in a very small number of works and many reports refers only to indomethacin; for these reasons it was not possible to make a reliable comparison between the safety profile of those drugs. Anyway, AEs were stated for 78 patients receiving indomethacin: the 54% of them suffered from an AE (mainly gastro-intestinal) and the 27% discontinued or interrupted the therapy.

### Short lasting unilateral neuralgiform headache attacks (SUNCT and SUNA)

Globally 56, studies were analyzed [124–179]. The most widely used treatment to control the excruciating and frequent attacks during active phases was lidocaine (36 patients), followed by prednisone (11 patients) and methylprednisolone (7 patients). To prevent the incoming of new active phases the most used treatments were: lamotrigine (84 patients), CBZ (78 patients), indomethacin (48 patients), gabapentin (48 patients) and topiramate (36 patients). All other treatments were used in less than 10 patients.

All these data are summarized in Table 1 (section C), data regarding statistical comparisons between the odds of responders and complete responders are summarized in Table 6 (acute treatments) and in Table 7 (prolonged treatments). Data regarding treatments used in less than 5 patients are reported in Additional file 1: Table S1 -section C.

**Table 6:**
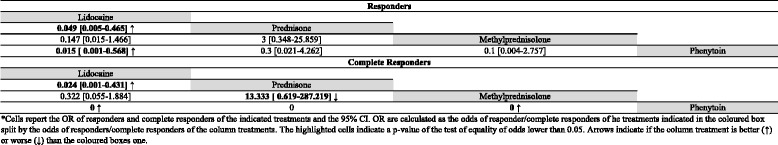
statistical comparisons between the odds of responders and complete responders of acute treatment for SUNCT and SUNA*

*Cells report the OR of responders and complete responders of the indicated treatments and the 95% CI. OR are calculated as the odds of responder/complete responders of he treatments indicated in the coloured box split by the odds of responders/complete responders of the column treatments. The highlighted cells indicate a *p*-value of the test of equality of odds lower than 0.05. Arrows indicate if the column treatment is better (↑) or worse (↓) than the coloured boxes' ones

**Table 7:**
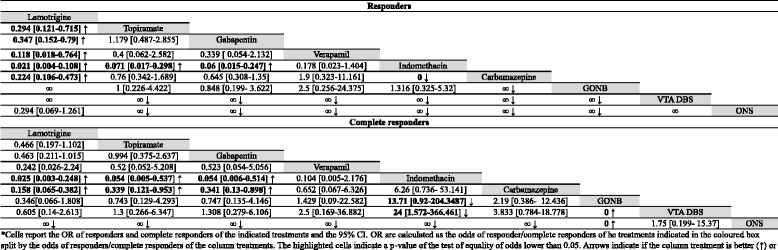
statistical comparisons between the odds of responders and complete responders of prolonged treatments for SUNCT and SUNA*

*Cells report the OR of responders and complete responders of the indicated treatments and the 95% CI. OR are calculated as the odds of responder/complete responders of he treatments indicated in the coloured box split by the odds of responders/complete responders of the column treatments. The highlighted cells indicate a *p*-value of the test of equality of odds lower than 0.05. Arrows indicate if the column treatment is better (↑) or worse (↓) than the coloured boxes' ones

#### Effectiveness

##### Acute treatments

Lidocaine, prednisone, methylprednisolone, phenytoin, celecoxib, superior trigeminal nerve blockade (STGB) and HDBS were considered for the management of exacerbation in SUNCT and SUNA. Lidocaine was effective in the 94% of patients, of which 80% of them were completely pain-free. Lidocaine has a significantly higher odds of responders than prednisone (*p* < 0.001) and phenytoin (*p* = 0.001), but comparable to methylprednisolone (*p* = 0.058). The same trend was seen for the odds of pain-free patients: lidocaine has an odds of complete responders significantly higher than prednisone (*p* = 0.002) and phenytoin (*p* < 0.001), but comparable to methylprednisolone (*p* = 0.1797). Methylprednisolone has significantly higher odds of complete responders than phenytoin (*p* = 0.0384). All comparisons are reported in Table 6. All other treatments were used upon less than 5 patients and weren’t pooled in the statistical analysis.

##### Prolonged treatments

Lamotrigine, topiramate, gabapentin, verapamil, indomethacin, CBZ, GONB, ventral tegmental area deep brain stimulation, ONS, clonazepam, HDBS, OnabotA, baclofen, pregabalin, gamma-knife radiosurgery of the trigeminal nerve, nifedipine, fentanyl, lithium, methysergide, zonisamide, lomerizine and STGB were those drugs used for the prevention of new active phases. The last 12 were not pooled in the statistical analysis due to the poor number of patients who tried them.

Lamotrigine has an odds of responders significantly higher than topiramate (*p* = 0.004), even if the odds of complete responders were comparable (*p* = 0.074). Lamotrigine has also a higher odds of responders (*p* = 0.008) and complete responders (*p* = 0.0487) than gabapentin and, moreover, than indomethacin, verapamil and CBZ (all *p*-value < 0.001).

Indomethacin has an odds of responders lower than topiramate, gabapentin, CBZ, VTA DBS and ONS (all *p* < 0.001). Ventral tegmental area deep brain stimulation and ONS have an odds of responders significantly higher than the ones of all other treatments despite lamotrigine (all *p*-values < 0.001).

Considering pain-free patients, indomethacin has a lower odds than lamotrigine, topiramate, gabapentin, GONB, VTA DBS and ONS (all *p*-values < 0.001). ONS has an odds of complete responders higher than all other treatments. All comparisons are reported in Table 7.

#### Safety

##### Acute treatments

Since the only reported AEs were for IV lidocaine, no statistical comparisons were made for short-term treatment drugs. Anyway, safety profile of IV or SC Lidocaine was stated for 36 patients, 13 of which suffered from a mild AE and 6 from an AE causing the discontinuation of therapy.

##### Prolonged treatments

According with the low number of signaled AEs, verapamil and indomethacin were excluded from the statistical analysis. Lamotrigine has more AEs than gabapentin (*p* = 0.039), but no differences were noted for the AEs causing the stop or the reduction of therapy (*p* = 0.232). No differences were found in the proportion of AEs between lamotrigine and CBZ (*p* = 0.311), but a tendency in a higher number of AEs causing the discontinuation or the modification of therapy was seen for CBZ (*p* = 0.06). Topiramate has a higher number of AEs than gabapentin (*P* = 0.002), but a similar occurrence of severe AEs. Topiramate has also the same proportion of AEs than CBZ and the same number of complete responders. Gabapentin was absolutely the safest drug, showing also a lower number of AEs than CBZ (*P* = 0.01). Because of the poor number of AEs causing the discontinuation or the modification of therapy, data regarding the comparison of their proportion between the different treatments were not shown in the previous Table.

## Discussion

### General considerations

Due to the infrequent diagnosis of these conditions, only case-reports or small case-series were found in literature and this strongly limits the reliability of the analysis. In many articles responders are not so well identifiable and in a very few ones the partial response was clearly described in terms of reduction of headache frequency, intensity or both, making almost impossible a comparison between the activity of different drugs on these aspects of pain. Treatment safety profile is hard to study too, primarily due to the sporadic report of AEs.

### Hemicrania continua (HC)

The first choice treatment for HC is indomethacin: for the management of recurrent exacerbations indomethacin should be the first choice drug, according with the higher effectiveness than all other treatments (see Table 2), which should be reserved to patients who don’t tolerated indomethacin. SONB has a similar proportion of responders but the lower odds of pain-free patients suggest that this technique is worse and more effective in diminishing pain rather than abolishing it [180]. It should also be considered that SONB has been tested only in a smaller number of patients than indomethacin and currently the experience on the use of these techniques is scarce, both for long-term availability (mean follow-up time = 93 days-data not show) and AEs profile. Celecoxib has an odds of responders lower than indomethacin but higher than GONB and an odds of complete responders higher than GONB and SONB, so it appears a better therapeutical approach than the last two in patients who don’t tolerate indomethacin. Piroxicam is comparable to celecoxib in terms of effectiveness, mirroring a similar action, as also stated by other studies [181]. GONB and MONB usefulness in relieving HC exacerbations seems to be negligible, like the usefulness of those treatments available for CH attacks, like SC sumatriptan and oxygen inhalation. This confirms that, despite the clinical over-lapping of HC and CH, the underlying pathogenetic mechanisms should be different, thus justifying a different pharmacological response [182]. HC management on long-time periods is unscheduled, but medications have been introduced trying to prevent pain recurrence. The prolonged use of drugs which were effective exacerbation control is a common practice and drugs like indomethacin, piroxicam and celecoxib are frequently used in HC patients outside active phases, even for many months: in our sample the duration of indomethacin assumption ranged between 5 and 1440 days, whereas from 18 to 540 for celecoxib. For piroxicam those data were not available, but its use for “many months” was reported in 5 patients out of 7. The stoppage of these drugs was due to AEs, mainly gastro-intestinal (GI), in the 70% of cases. The development of serious AEs is the main reason for which indomethacin, piroxicam and celecoxib should not be continued for many months outside exacerbations, even if the dose is titrated to the lowest possible or a preventive therapy with a proton pump inhibitor is started. SONB and GONB were both used even for the prevention of HC exacerbations, but GONB seems of no effect and SONB has a low odds of pain-free patients, denoting a partial action. The incoming of GI AEs and the low effectiveness of GONB and SONB impose the use of other drugs to control pain.

Gabapentin, topiramate, melatonin and OnabotA seems to be comparable in terms of effectiveness even if, considering the *p*-values of these comparisons (*p* = 0.063), a better action for gabapentin and topiramate than melatonin should be hypothesed. ONS should be a reliable option besides pharmacological techniques, as also confirmed from a recently published statement from the European Headache Federation [183]. The usefulness of verapamil in HC is scarce, since it has a lower odds of responders than indomethacin, OnabotA, topiramate, ONS and gabapentin and an odds of complete responders lower than all other treatments, except the non-pharmacological ones and melatonin.

The question on the tolerability of these treatments remains open and the unfair data about AEs make any comparison doubtful. Anyway, from the available literature, celecoxib and piroxicam should have a similar AEs profile than indomethacin with an even higher risk of cardiovascular side-effects with celecoxib [184], but a lower risk of renal AEs according to its higher COX-2 selectivity [185]: celecoxib and other COX-2 selective NSAIDs should be avoided with cardiovascular co-morbidities, but should be chosen after indomethacin in patients with renal diseases of with gastro-intestinal co-morbidities.

### Paroxysmal hemicrania (PH)

PH is another member of the so-called indomethacin-responsive headaches [1] and, in fact, indomethacin is undoubtedly the best treatment even for this condition. The activity of other treatments is low both for the acute treatment and for the prolonged one. Piroxicam emerges as the best treatment besides indomethacin for exacerbations management, according to the higher odds of pain-free patients than oxygen and sumatriptan. The usefulness of this last two drugs is almost null and this confirms once again the differences in TACs’ pathogenesis besides their clinical similarity [182]. Even when used for PH control outside active phases indomethacin is the most effective treatment. Even so, since PH is frequently chronic and indomethacin assumption for long periods of time may cause a wide range of AEs, this usually lead to the discontinuation of therapy in about 27% of cases. This imposes the use of different treatments to control the pain, but other tested drugs seems to be of little use with the most effective being rofecoxib, which has been retired from the international market because of its cardiac side-effects [186]. Piroxicam seems to be the most effective treatment other than indomethacin, even if the possibility of having GI AEs remains [187] and, like indomethacin, its use should be avoided for long periods of time. Since the hypothesized overlapping between PH and migraine pathogenesis [15], two well-known migraine prophylaxis such as topiramate and amitriptyline have been tried for PH, with comparable and moderate results. Topiramate and amitriptyline are also comparable to piroxicam and verapamil in terms of effectiveness, even though the latter shows a not-significant higher odds of responders and complete responders. CBZ usefulness seems to be low and the null number of complete responders should discourage its use for PH management.

### Short lasting unilateral neuralgiform headache attacks (SUNCT and SUNA)

To stop SUNCT and SUNA exacerbations, lidocaine (intravenously or subcutaneously) seems to be the most effective treatment and is now emerging as a novel option for chronic pain syndromes [188]. Its effectiveness is unquestionable, but paranoid idealization, depressive thoughts and cardiac arrhythmias were registered as AEs: this imposes the careful and shortest use of this drug only for the worst cases and the patient’s continuous monitoring with a 12-lead ECG registration and sequential blood pressure measurements during the treatment [189]. In our sample the time of use ranged between 2 to 10 days (data not shown). Steroids represent a less effective but safer options for stopping attacks, with methylprednisolone presenting a better action than prednisone, even if not significantly. As previously discussed for lidocaine, steroids should be given intravenously for the shortest time as possible: from literature it is well-known that they can have a wide range of AEs, which can be prevented by reducing the duration of infusion to the time necessary for the ceasing of painful exacerbations [190]. In our sample the mean time of infusion was 8 ± 4.32 days (data not shown) The usefulness of phenytoin should be considered negligible.

Lamotrigine is the best drug for the prevention of the incoming of new active phases, but seems to be more suitable in reducing attacks frequency rather than abolishing them completely: it has an odds of partial responders higher than all other drugs, but the odds of complete responders are comparable, with the exceptions of CBZ and indomethacin, which efficacy is scarce. Non-pharmacological techniques have an odds of responders comparable to lamotrigine and, moreover, ONS has even a higher odds of complete responders. Lamotrigine has also a similar AEs profile than other treatments except for gabapentin, confirming the available literature [191].

Verapamil, gabapentin and topiramate have similar effectiveness, with gabapentin showing a better AEs profile, even if the number of reported AEs is too poor to let a reliable comparison. CBZ appears less useful in treating SUNCT and SUNA than gabapentin and topiramate, according with the lower number of complete responders. Indomethacin usefulness in these conditions is sometimes reported, but should be considered as negligible: an occasional benefit of this drug in SUNCT or SUNA should rise the question of a diagnostic mistake with HC, PH or a secondary headache, imposing the reconsideration of the initial diagnosis, following scheduled diagnostic algorithms [192].

Recently, non-pharmacological techniques has gained importance in the treatment of these disorders, but the experience with these treatments is scarce and the long-term follow-up of patients is often lacking in many studies. From the available data ONS has emerged as the best technique and this result is in accordance with the findings in CH, were ONS is the only class-A evidence treatment for the American Headache Society (AHS) [193]. Moreover, even the European Headache Federation (EHF) has confirmed the effectiveness and safety of this technique in SUNCT and SUNA, pointing out that 4 patients out of 6 analyzed were nearly pain free with mild facial paresthesia as the principal AE [183].

From the reviewed literature, ONS has demonstrated an almost complete effectiveness and a good safety profile, but it has been tried only on 7 patients. Ventral tegmental area deep brain stimulation has shown a similar effectiveness, but adverse events were reported in the 100% of cases and should be reserved to the refractory cases. Finally, GONB appears to be less effective but also safer than the previous techniques and should be considered as a reliable alternative in patients with episodic forms.

**Fig. 2:**
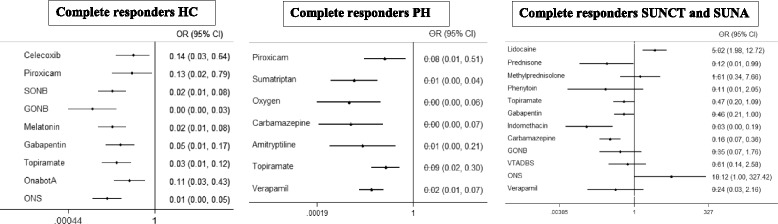
Odds ratios of complete responders. For HC and PH the referral treatment is indomethacin. For SUNCT and SUNA the referral treatment is lamotrigine. If the whole 95% CI of the OR is lower than 1, the referral treatments is better than the reported one

In Fig. 2 the ORs of complete responders and the relative IC95% are visually summarized for all diseases. ORs are calculated as the odds of pain-free patients for the indicated treatments split by the odds of pain-free patients for the most used treatment for every disease.

## Conclusion

PH, HC, SUNCT and SUNA represent a hard challenge for clinicians who work in headache or pain fields. Moreover, their infrequence makes difficult to study the pathogenesis of these conditions, as well as design well-done RCPCT for new drugs. From the review of the available literature indomethacin emerges as the best treatment for HC and PH, while other drugs like celecoxib, topiramate and gabapentin may be useful. SUNCT and SUNA should be managed with intravenous steroids or lidocaine in the worst cases and for short periods of time, with a subsequent change for preventive treatment to lamotrigine or ONS.

In conclusion, it should be highlighted that further studies are required to implement guidelines to treat the disease and to discover new effective and safe therapies for these conditions.

## Additional file

Additional file 1: Treatment options for HC, PH and SLUNHA used in less than 5 patients. (DOC 108 kb)

## Abbreviations

AE: Adverse event
AHS: American Headache Society
CBZ: Carbamazepine
EHF: European Headache Federation
GONB: Great occipital nerve blockade
HC: Hemicrania continua
ICHD-III-beta: International classification of headache disorders-3rd edition, beta version
IHS: International Headache Society
IV: Intravenous
MONB: Minor occipital nerve blockade
NSAIDs: Non-steroidal anti-inflammatory drugs
OnabotA: onabotulinumtoxinA
ONS: Occipital nerve stimulation
OR: Odds ratio
PH: Paroxysmal hemicrania
RCPCT: Randomized clinical placebo-controlled trials
SLUNHA: Short-lasting unilateral neuralgiform headache attacks
SON: Supra-orbital nerve
SONB: Supraorbital nerve blockade
STGB: Superior trigeminal ganglion blockade
