# Supplementary material for: Therapeutical approaches to paroxysmal hemicrania, hemicrania continua and short lasting unilateral neuralgiform headache attacks: a critical appraisal
Source: J Headache Pain. 2017 Jul 20;18(1):71. doi: 10.1186/s10194-017-0777-3 (PMC5519518; doi:10.1186/s10194-017-0777-3)
Supplement: Additional file 1: — Treatment options for HC, PH and SLUNHA used in less than 5 patients. (DOC 108 kb) [file 10194_2017_777_MOESM1_ESM.doc]

| **Table S1: Treatment options for HC, PH and SLUNHA used in less than 5 patients** | | | | | | | | |
| --- | --- | --- | --- | --- | --- | --- | --- | --- |
| **Treatment** | **Number of patients** | **Mean Dosage ±SD* or procedure for non-pharmacological treatments** | **Route of administration** | **Responders**  **Proportion**  **% [95% CI]** | **Complete responders**  **Proportion**  **% [95% CI]** | **AE Proportion**  **% [95% CI]** | **AE causing the stoppage or reduction of therapy proportion**  **% [95% CI]** | **References** |
| ***Section A- Hemicrania continua*** | | | | | | | | |
| *Acute treatments* | | | | | | | | |
| Ibuprofen | 3 | 600 | OS 100% | 1/3  33 | 0/3  0 | - | - | [38,68,69] |
| Methylprednisolone | 2 | 665±200  [500-1000] | IV 100% | 2/2  100 | 2/2  100 | - | - | [8] |
| DRGB | 2 | 9 cc of 1% lidocaine with  40 mg triamcinolone |  | 2/2  100 | 2/2  100 | - | - | [35] |
| Sphenopalatine ganglion block | 1 | 9 cc of 1% lidocaine with 40 mg triamcinolone |  | 1/1  100 | 1/1  100 | 1/1  100 | 1/1  100 | [35] |
| Ergotamine | 1 | 2 | OS 100% | 0 | 0 | - | - | [32] |
| *Prolonged treatments* | | | | | | | | |
| Acemethacin | 3 | 90 | OS 100% | 3/3  100 | 3/3  100 | - | - | [51] |
| Amytriptiline | 2 | 50 | OS 100% | 0/2  0 | 0/2  0 | - | - | [49,55] |
| DRGB | 2 | 9 cc of 1% lidocaine with  40 mg triamcinolone |  | 2/2  100 | 2/2  100 | - | - | [35] |
| Sphenopalatine ganglion block | 1 | 9 cc of 1% lidocaine with 40 mg triamcinolone |  | 1/1  100 | 1/1  100 | 1/1  100 | 1/1  100 | [35] |
| Valproate | 1 | 750 | OS 100% | 1/2  50 | 1/2  50 | - | - | [43] |
| Lithium | 1 | 350 | OS 100% | 0/2  0 | 0/2  0 | - | - | [43] |
| Troclear injection of Triamcinolone | 1 | 4 | SC 100% | 1/1  100 | 1/1  100 | - | - | [34] |
| Fentanyl | 1 | 50^ | TD 100% | 1/1  100 | 0/1  0 | - | - | [28] |
| Tilidine | 1 | 300 | OS 100% | 1/1  100 | 0/1  0 | - | - | [46] |
| ***Section B- Paroxysmal hemicrania*** | | | | | | | | |
| *Acute treatments* | | | | | | | | |
| Prednisone | 4 | 63±26  [40-100] | OS 100% | 1/4  25 [10-40] | 1/4  25 [10-40] | - | - | [38,78,91,98] |
| Valdecoxib | 1 | 20 | OS 100% | 0/1  0 | 0/1  0 | - | - | [96] |
| Etoricoxib | 1 | 120 | OS 100% | 1/1  100 | 1/1  100 | - | - | [96] |
| Naproxen | 1 | 550 | OS 100% | 1/1  100 | 0/1  0 | 1/1  100 | 1/1  100 | [80] |
| Betahmetasone | 1 | 4 | OS 100% | 1/1  100 | 0/1  0 | - | - | [109] |
| Methylprednisolone | 1 | 60 | IM 100% | 1/1  100 | 0/1  0 | - | - | [98] |
| HDBS | 1 | 1.5 |  | 1/1  100 | 1/1  100 | 0/1  0 | 0/1  0 | [123] |
| Sphenopalatine ganglion blockade | 1 |  |  | 1/1  100 | 0/1  0 | 0/1  0 | 0/1  0 | [101] |
| *Prolonged treatments* | | | | | | | | |
| Propranolol | 3 | 93±23  [80-120] | OS 100% | 0/3  0 | 0/3  0 | - | - | [78,83,90] |
| ASA | 3 | 500 | OS 100% | 2/3  67 [56-78] | 1/3  33 [21-42] | 0/1  0 | 0/1  0 | [104,116,121] |
| Lithium | 3 | 1000±173  [900-1200] | OS 100% | 0/3  0 | 0/3  0 | - | - | [91,98,101] |
| Ergotamine | 2 | 2 | OS 100% | 0/2  0 | 0/2  0 | - | - | [92,103] |
| Dipyrone | 2 | 1.75±0.35  [1.5-2] | OS 100% | 1/2  50 [0-90] | 0/2  0 | - | - | [73,116] |
| Valproate | 2 | 450±212  [300-600] | OS 100% | 1/2  50 [0-90] | 0/2  0 | - | - | [93,111] |
| Acetazolamide | 2 | 750 | OS 100% | 2/2  100 | 2/2  100 | - | - | [91,107] |
| Baclofen | 1 | 30 | OS 100% | 1/1  100 | 1/1  100 | - | - | [110] |
| Phenytoin | 1 | 300 | OS 100% | 1/1  100 | 1/1  100 | - | - | [110] |
| Methysergide | 1 | 24 | OS 100% | 0/0  0 | 0/0  0 | - | - | [102] |
| Doxepine | 1 | 20 | OS 100% | 0/0  0 | 0/0  0 | - | - | [85] |
| Flunnarizine | 1 | 5 | OS 100% | 0/1  0 | 0/1  0 | - | - | [93] |
| Gabapentin | 1 | 900 | OS 100% | 0/1  0 | 0/1  0 | - | - | [100] |
| Betahmetasone | 1 | 4 | OS 100% | 1/1  100 | 0/1  0 | - | - | [109] |
| Methylprednisolone | 1 | 60 | IM 100% | 1/1  100 | 0/1  0 | - | - | [98] |
| OnabotulinumtoxinA | 1 | 30^^ | SC 100% | 1/1  100 | 1/1  100 | - | - | [122] |
| HDBS | 1 | 1.5 |  | 1/1  100 | 1/1  100 | 0/1  0 | 0/1  0 | [123] |
| Sphenopalatine ganglion blockade | 1 |  |  | 1/1  100 | 0/1  0 | 0/1  0 | 0/1  0 | [101] |
| **Section C- Short lasting unilateral neuralgiform headache attacks** | | | | | | | | |
| *Acute treatments* |  |  |  |  |  |  |  |  |
| Celecoxib | 2 | 200 | OS 100% | 1/2  50 | 0/2  0 | - | - | [124,139] |
| Superior Trigeminal ganglion blockade with buprenorfphine | 1 | 0.03 | Ganglion blockade | 1/1  100 | 1/1  100 | - | - | [161] |
| HDBS 1.8 V, pulse width180 Hz, 60 μs | 4 | Amplitude: 1.8V  Frequency: 180 Hz  Pulse width: 60μs | Hypothalamic stimulation |  |  |  |  | [176-179] |
| *Prolonged treatments* |  |  |  |  |  |  |  |  |
| Clonazepam | 4 | 1.75 | OS 100% | 0/4  0 | 0/4  0 | - | - | [124,164,165,171] |
| HDBS 1.8 V, pulse width180 Hz, 60 μs | 4 | Amplitude: 1.8V  Frequency: 180 Hz  Pulse width: 60μs | Hypothalamic stimulation |  |  |  |  | [176-179] |
| OnabotulinumtoxinA | 2 | 5-10^^ | SC | 2/2  100 | 2/2  100 | - | - | [126,153] |
| Pregabalin | 2 | 112.5 | OS 100% | 0/0  0 | 0/0  0 | - | - | [126,157] |
| Baclofen | 2 | 30 | OS 100% | 1/2  50 | 1/2  50 | - | - | [124,133] |
| Gamma-knife radiosurgery of the trigeminal nerve (90 Gy) | 2 | 90^^^ | Radiosurgery | 1/2  50 | 0/2  0 | - | - | [138] |
| Nifedipine | 1 | 2 | OS 100% | 1/1  100 | 0/1  0 | - | - | [131] |
| Fentanyl | 1 | 50 | TD 100% | 1/1  100 | 0/1  0 | - | - | [129] |
| Lithium | 1 | 900 | OS 100% | 0/0  0 | 0/0  0 | - | - | [161] |
| Methysergide | 1 | 4 | OS 100% | 1/1  100 | 0/1  0 | - | - | [139] |
| Zonisamide | 1 | 300 | OS 100% | 1/1  100 | 1/1  100 | 1 | 0 | [165] |
| Lomerizine | 1 | 10 | OS 100% | 1/1  100 | 1/1  100 |  |  | [155] |
| Superior Trigeminal ganglion blockade with buprenorfphine | 1 | 0.03 | Ganglion blockade | 1/1  100 | 1/1  100 | - | - | [161] |

*For non-pharmacological procedures the method used has been reported. Drug dosages are in mg/day if not otherwise specified. ^μg,^^UI,^^^Gy
